# Amyloid-β misfolding as a plasma biomarker indicates risk for future clinical Alzheimer’s disease in individuals with subjective cognitive decline

**DOI:** 10.1186/s13195-020-00738-8

**Published:** 2020-12-24

**Authors:** Julia Stockmann, Inge M. W. Verberk, Nina Timmesfeld, Robin Denz, Brian Budde, Julia Lange-Leifhelm, Philip Scheltens, Wiesje M. van der Flier, Andreas Nabers, Charlotte E. Teunissen, Klaus Gerwert

**Affiliations:** 1 Competence Center for Biospectroscopy, Center for Protein Diagnostics (PRODI), Ruhr-University Bochum, Bochum, Germany; 2 Department of Biophysics, Ruhr University Bochum, Faculty of Biology and Biotechnology, Bochum, Germany; 3 Neurochemistry Laboratory, Department of Clinical Chemistry, Amsterdam Neuroscience, Vrije Universiteit Amsterdam, Amsterdam UMC, Amsterdam, The Netherlands; 4 Alzheimer Center Amsterdam, Department of Neurology, Amsterdam Neuroscience, Vrije Universiteit Amsterdam, Amsterdam UMC, Amsterdam, The Netherlands; 5 Ruhr University Bochum, Department of Medical Informatics, Biometry and Epidemiology, Bochum, Germany

**Keywords:** Alzheimer’s disease, Amyloid beta (Aβ), Blood plasma, Risk stratification, Structure biomarker

## Abstract

**Background:**

We evaluated Aβ misfolding in combination with Aβ_42/40_ ratio as a prognostic tool for future clinical progression to mild cognitive impairment (MCI) or dementia due to Alzheimer’s disease (AD) in individuals with subjective cognitive decline (SCD).

**Methods:**

Baseline plasma samples (*n* = 203) from SCD subjects in the SCIENCe project and Amsterdam Dementia Cohort (age 61 ± 9 years; 57% male, mean follow-up time 2.7 years) were analyzed using immuno-infrared-sensor technology. Within 6 years of follow-up, 22 (11%) individuals progressed to MCI or dementia due to AD. Sensor readout values > 1646 cm^− 1^ reflected normal Aβ folding; readouts at ≤ 1646 cm^− 1^ reflected low and at < 1644 cm^− 1^ high misfolding. We used Cox proportional hazard models to quantify Aβ misfolding as a prognostic biomarker for progression to MCI and dementia due to AD. The accuracy of the predicted development of MCI/AD was determined by time-dependent receiver operating characteristic (t-ROC) curve analyses that take individual follow-up and conversion times into account. Statistical models were adjusted for age, sex, and APOEε4 status. Additionally, plasma Aβ_42/40_ data measured by SIMOA were statistically analyzed and compared.

**Results:**

All 22 patients who converted to MCI or AD-dementia within 6 years exhibited Aβ misfolding at baseline. Cox analyses revealed a hazard ratio (HR) of 19 (95% confidence interval [CI] 2.2–157.8) for future conversion of SCD subjects with high misfolding and of 11 (95% CI 1.0–110.1) for those with low misfolding. T-ROC curve analyses yielded an area under the curve (AUC) of 0.94 (95% CI 0.86–1.00; 6-year follow-up) for Aβ misfolding in an age, sex, and APOEε4 model. A similar model with plasma Aβ_42/40_ ratio yielded an AUC of 0.92 (95% CI, 0.82–1.00). The AUC increased to 0.99 (95% CI, 0.99–1.00) after inclusion of both Aβ misfolding and the Aβ_42/40_ ratio.

**Conclusions:**

A panel of structure- and concentration-based plasma amyloid biomarkers may predict conversion to clinical MCI and dementia due to AD in cognitively unimpaired subjects. These plasma biomarkers provide a noninvasive and cost-effective alternative for screening early AD pathological changes. Follow-up studies and external validation in larger cohorts are in progress for further validation of our findings.

**Supplementary Information:**

The online version contains supplementary material available at 10.1186/s13195-020-00738-8.

## Background

Biomarkers indicating Alzheimer’s disease (AD) in cognitively unimpaired individuals are essential for future therapeutic approaches [1]. In clinical trials, amyloid-β (Aβ) positron emission tomography (Aβ-PET) is used to visualize amyloid in the brain [2–6]. Aβ-PET targets fibrillary amyloid plaques; however, the technique is costly [3]. Since the increased deposition of Aβ_42_ in amyloid plaques is strongly associated with its decreased CSF concentration [7], the Aβ_42_ concentration in CSF is widely used as a fluid biomarker for AD [8–12]. Unfortunately, CSF necessitates invasive lumbar puncture, thereby limiting its use [13]. Therefore, there is an urgent need to identify blood-based biomarkers for AD. After several years of controversial results in cohort studies [14], the utility of the Aβ_42/40_ ratio as a noninvasive blood plasma biomarker has recently shown promising results, as it correlates with brain amyloid pathology [15–17]. Additionally, recent studies have suggested prognostic value for plasma Aβ_42/40_ [16, 18–20]. This is due to the novel methodological improvements that allow sensitive analysis of plasma Aβ concentrations by immunoassays such as SIMOA [16, 21] or mass spectrometry [15, 17, 22].

Complementary to these measurements of Aβ concentrations in plasma indicating indirectly the amyloid plaque formation, Aβ misfolding in blood plasma might serve as an additional biomarker for an early disease stage [23–26]. In vitro, it was shown that plaques are formed by initial misfolding of Aβ from a predominantly monomeric alpha-helical and disordered structure to a β-sheet-enriched secondary structure. This structural change triggers and initiates Aβ oligomerization and aggregation to much larger fibrils on the nanometer scale [27–32]. Since misfolding of Aβ causing peptide aggregation and plaque formation is believed to be one of the initial events in AD development and starts 15–20 years before clinical symptoms occur [4, 27, 33], it is conceivable that Aβ misfolding might be one of the earliest detectable events in AD pathogenesis. Therefore, Aβ misfolding might be a promising risk marker to identify high-risk individuals in the very early stages of the disease.

Aβ misfolding can be monitored using an immuno-infrared sensor (iRS), which measures the frequency of the C=O stretching vibration of the Aβ backbone [23, 34]. This vibration causes the amide I absorbance band, which in turn gives information about the secondary structure distribution of all Aβ isoforms [23].

We have recently validated Aβ misfolding as a structure biomarker in plasma for probable AD (prospective Essen cohort) [24], for prodromal AD (BioFINDER) [25], and for preclinical cognitively unimpaired AD subjects (population-based ESTHER cohort) [25]. Including APOEε4 as a risk factor, early AD pathology could be identified with an AUC of 0.84 as early as 14 years before the clinical diagnosis of AD [35]. Remarkably, consistent cutoffs have been observed across all studies with a general threshold of < 1644 cm^− 1^, indicating high misfolding in individuals with dementia due to AD. A second upper threshold of > 1646 cm^− 1^ was recently introduced to further differentiate between already low misfolding and a “normal” Aβ secondary structure distribution, as observed in individuals without dementia due to AD [26].

Here, we analyzed baseline plasma samples of individuals with subjective cognitive decline (SCD) from the memory clinic-based SCIENCe study and the Amsterdam Dementia Cohort, to further explore the potential of the structure-based plasma biomarker and the prognostic value in cognitively unimpaired individuals [16, 36–38]. However, SCD individuals have a higher risk of progression to dementia compared to cognitively unimpaired individuals without subjective complaints [39]. Furthermore, we explored, whether the combination of structure-based and concentration-based Aβ_42/40_ plasma biomarkers could increase the prognostic performance in a panel including other covariates to predict clinical progression to MCI or AD-dementia.

## Methods

### Study cohort

A total of 236 baseline EDTA plasma samples were received from individuals in the ongoing Amsterdam Dementia Cohort and SCIENCe project [36–38], which was a subset of the study population described in detail by Verberk et al. [16]. The inclusion criteria for the current study comprised a baseline diagnosis of SCD received within 0.5 years of plasma sampling, the plasma sample had to be nonhemolytic, at least one follow-up visit was available, and follow-up diagnosis confirmed either SCD or conversions to MCI or AD-dementia. Eight subjects progressed to non-AD-dementia and were excluded. Only the subjects with baseline data on possible covariates, such as age, sex, and APOEε4 status, were included. The total number of subjects diagnosed with SCD eligible for inclusion in the current study was 203.

All subjects were referred to the Alzheimer Center, Amsterdam between 2000 and 2016 because of cognitive complaints, where they were thoroughly screened for their neurological, physical, and neuropsychological functioning. Furthermore, brain magnetic resonance imaging, electroencephalography, Aβ_1–42_ CSF biomarker analysis by Innotest ELISAs (Fuijirebio, Ghent, Belgium), and APOEε4 genetic screening by polymerase chain reaction were performed. CSF Aβ_42_ levels were dichotomized into positive (< 813 pg/mL) or negative (> 813 pg/mL) [40]. In a multidisciplinary consensus meeting, the label “SCD” was assigned, since the subject’s report of cognitive worsening could not be objectified by any of the clinical or cognitive tests performed, and the criteria for MCI, dementia, or other medical conditions possibly explaining the perceived cognitive decline were not met [41]. Written informed consent to use medical data and biomaterials for research purposes was in place, in accordance with ethical approval from the VU University Medical Center based on the Helsinki Declaration (seventh revision).

### Clinical follow-up

After the baseline visit, individuals were regularly examined, including repeated neurological, physical, and neuropsychological assessments. Clinical diagnosis was re-evaluated after each visit by clinical consensus using valid diagnostic criteria [16, 36–38]. Clinical progression was defined as the change in diagnosis to MCI [42, 43] or AD-dementia [44, 45], and the time point of progression was defined as the visit date corresponding to when the diagnosis first changed; when subjects first progressed to MCI and later to dementia, the time of the MCI diagnosis was used as the time point of clinical progression.

**Fig. 1:**
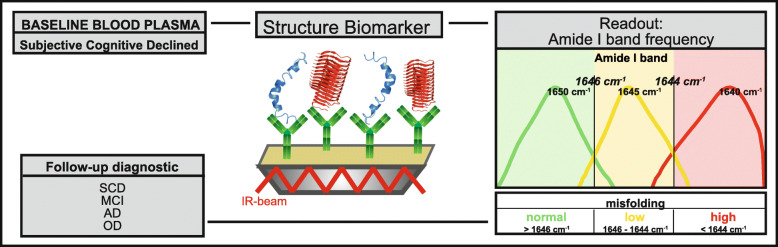
Schematics of the study workflow. Baseline blood plasma samples from 203 individuals with subjective cognitive decline (SCD) were analyzed. Plasma samples were circulated over the iRS surface. Antibodies bound on the surface catch all isoforms of Aβ out from the plasma. The readout is the infrared frequency of the amide I band of all antibody-bound Aβs. This frequency is indicative of the Aβ secondary structure distribution in plasma. Frequencies above 1646 cm^− **1**^ in green represent a normal secondary structure distribution, as found in healthy subjects. Frequencies below 1646 cm^− **1**^ indicate low misfolding (yellow), and those below 1644 cm^− **1**^ (red) indicate high misfolding as observed in AD-affected individuals. The predictive performance of the structure biomarker was validated with clinical diagnosis at 6 years of follow-up. SCD = subjective cognitive decline; MCI = mild cognitive impairment; AD = Alzheimer’s disease; OD = other dementia

### Blood plasma collection, processing, and preanalytics

Blood for preparation of EDTA plasma was drawn through venipuncture at the Amsterdam UMC, VU University Medical Center. Plasma tubes were centrifuged at 1800×*g* for 10 min within 1 h of collection, and plasma was aliquoted in 0.5-mL portions in polypropylene tubes and stored at − 80 °C. Samples were first thawed at the Amsterdam UMC for the quantitative determination of plasma concentrations of Aβ_1–40_ and Aβ_1–42_ with the SIMOA Human Neurology 3-Plex A assay kit (Quanterix, Lexington, MA) using the SIMOA HD-1 analyzer [16]. Thereafter, 200 μl of plasma per subject was shipped from Amsterdam to Bochum on dry ice.

### Antibody and plasma preparation

For the iRS, we used the monoclonal antibody HJ5.1 as capture antibody, which specifically binds to all structural isoforms of Aβ (see supplementary Fig. S-1). The antibody HJ5.1 was developed, produced, and validated by Holtzman et al. from Washington University (Washington, USA). The antibody HJ5.1 has also been used for Aβ extraction in other assays by different groups [22]. We received the hybridoma cell line for in-house production of HJ5.1. Because of the low human anti-mouse antibody and interference of HJ5.1 with other human IgGs in our assay, we established a standardized sample preprocessing protocol for IgG removal from the plasma sample. In general, the depletion procedure comprised two different strategies for IgG removal. In strategy 1, the Pierce™ Spin column (0.5 ml volume, Thermo Scientific, Germany) was charged with 500 μl of Pierce™ Protein G Agarose resin (Thermo Scientific, Germany) and blocked with 150 μl of 1% (w/v) casein solution for 2 h. Afterwards, the column was washed with 3 ml of phosphate-buffered saline (PBS; pH 7.4) buffer. Subsequently, 100 μl of plasma sample was loaded on the prepared column and incubated overnight at 4 to 6 °C under light shaking conditions. As a final step, the remaining IgGs were eliminated from the plasma sample with magnetic beads. To this end, the plasma sample was eluted from the column and spiked with magnetic beads that are covalently linked with Protein A and Protein A/G and Protein G. The admit volume of the magnetic beads was 50 μl of each kind of bead. The plasma sample was incubated for at least 3 h under light shaking at 4–6 °C. Afterwards, the magnetic beads were completely removed from the sample via a magnetic stand, and the sample was immediately measured. To ensure that there is no antibody interference signal during sample analysis in our assay, all IgG-depleted plasma samples (strategy 1) simultaneously underwent quality control. Therefore, the surface of a separate sensor element was covered with antibody binding protein A employing silane chemistry. Such a sensor surface is highly specific to detect the lowest amounts of residual IgG interference within the plasma sample. The procedure of this sensor modification is described in detail by Budde et al. [46]. If no antibody interference was detected, the quality control confirmed the complete IgG removal from the plasma sample; otherwise, if strategy 1 was insufficient to completely remove IgGs from the sample and antibody interference was observed by two characteristic antibody absorbance peaks at 1638 cm^− 1^ and 1685 cm^− 1^, we extended IgG removal of this plasma sample by an alternative double column depletion strategy (strategy 2). Therefore, the first step of the depletion process was performed via Pierce™ Protein G Agarose resin as described above (strategy 1) followed by a second column charged with 500 μl of Pierce™ Protein A Agarose resin (Thermo Scientific, Germany). The protein A agarose column was incubated overnight at 4 to 6 °C under light shaking conditions.

To validate that the depletion process had no effect on the secondary structure of Aβ and on the diagnostic performance of the iRS, we compared the results of single- and double-depleted plasma samples measured with HJ 5.1 with each other and with previous measurements from these patient samples, in which the monoclonal antibody A8978 (Sigma Aldrich, Germany) was used [24, 25]. Since this antibody does not show any interference with human antibodies, we do not have to perform IgG depletion of the samples. Therefore, these measurements can serve as controls to indicate whether the depletion affects the Aβ secondary structure distribution. Our results with antibody HJ5.1 were highly reproducible (Fig. S-2), independent of the depletion strategy, and highly comparable to the diagnostic results obtained using antibody A8978 (Fig. S-3). Hence, the depletion procedure for IgG removal from the plasma samples had no effect on the secondary structure of Aβ in our assay. Both IgG removal procedures guaranteed the elimination of any antibody interference signal in our assay. Importantly, strategy 2 also had no influence on the Aβ secondary structure distribution within the required timeline (supplementary Fig. S-2). Samples were immediately measured after depletion to avoid further sample handling and additional freeze-thaw cycles. However, sample age in general showed no influence on the iRS readout (supplementary Fig. S-4). If sufficient, strategy 1 was preferred because of time and cost constraints as well as to avoid the hurdles of sample preparation. In the present study, strategy 1 was employed for 80% of the samples, and strategy 2 was employed for 20% of the samples.

### Plasma analysis by the immuno-infrared sensor (iRS)

Baseline blood plasma samples were analyzed with our iRS [23] as schematically outlined in Fig. 1. The iRS has been validated in detail, including NHS silane generation and characterization, antibody batch-to-batch variation, antibody performance with synthetic Aβ, and standard reference CSF and blood plasma samples, matrix effects, lower and upper limits of quantification, assay selectivity, sample handling and documentation of zero background signals after Aβ immunodepletion. These procedures and protocols have been previously described in detail [23]. In short, the monoclonal antibody HJ5.1 (D. Holtzman, Washington) was covalently immobilized on the sensor surface by silane chemistry followed by surface blocking via a casein solution. By using a flow cuvette that housed the functionalized sensor surface, the plasma samples were circulated in a constant flow (1 ml/min) for 1 h to extract the total Aβ fraction. This step was followed by a washing step for 30 min with PBS buffer to remove unbound substances from the sensor surface. The binding signal of the sample stayed stable over circulation and washing time and did not result in any change in the Aβ structure during the analysis procedure (supplementary Fig. S-5). Importantly, the antibody HJ5.1 simultaneously binds to all structural isoforms of Aβ, including monomeric, oligomeric, and fibrillary Aβ. By difference spectroscopy, only the absorbance band of bound Aβ is elucidated, and the absorbance bands of all other components are subtracted. As a readout, the infrared frequency of the amide I absorbance band of the bound Aβ fraction was recorded. The amide I band records the absorbance of the secondary structure-sensitive C=O stretching vibration of the Aβ peptide backbone. Monomeric Aβ isoforms with alpha-helical and unstructured secondary structures show a maximum frequency at 1655 wavenumbers (cm^− 1^), whereas β-sheet enriched structures have a maximum at 1624 cm^− 1^ [23]. Misfolding from predominantly monomeric alpha-helical and disordered Aβ in healthy individuals to increased β-sheet enriched structures in individuals with AD shifts the frequency down. iRS readout values < 1644 cm^− 1^ were indicative of increased high misfolding, and values > 1646 cm^− 1^ reflected normal folding. Hence, values between ≥ 1644 and ≤ 1646 cm^− 1^ indicated a slightly increased misfolding level, defined as low misfolding. The threshold of 1644 cm^− 1^ (± 1 cm^− 1^) was empirically determined in [25]. The second upper threshold at 1646 cm^− 1^ (± 1 cm^− 1^) was recently introduced in Nabers et al. in the context of a two-step diagnostic workflow [26]. With this threshold, we identified individuals with a largely increased likelihood of disease onset. Plasma samples were analyzed at baseline, during which all individuals were clinically diagnosed as cognitively unimpaired.

### Statistical analysis

In the statistical analyses, we did not differentiate between subjects who converted to either MCI or dementia due to AD and included them in one converter group. For the description of groups, mean ± SD were used for continuous variables and absolute number and percentage for categorical variables. Comparisons between groups were performed with nonparametric tests (Wilcoxon rank-sum test). *p* values less than 0.05 were considered significant. Since the patient-related follow-up period differed between the subjects within this longitudinal study, we applied statistical models that take event times (including censoring) into account. We used the Cox proportional hazard model, both unadjusted and adjusted for relevant covariates (sex, age, and APOEε4), to calculate the risk of clinical disease progression. Additionally, we used time-dependent receiver operating characteristic (t-ROC) curves to calculate the diagnostic accuracy for non-converted SCD vs. follow-up MCI or AD-dementia based on baseline biomarkers according to Uno et al. [47]. This method takes the interindividual differences in follow-up and conversion times into account. The t-ROC results could be interpreted in a way similar to that for usual ROC curves.

Survival curves were plotted to visualize the difference in conversion rates between subjects with normal folding and those low and high misfolding (unadjusted curves). For sensitivity analysis (for results, see appendix), similar Cox proportional hazard models were fitted for all available participants. For sensitivity analysis, patients with progression to other dementia were censored at the time of progression.

Analyses were performed with Origin 2017, MATLAB 2015, and R, version 3.5.1 using the packages “coin,, “survival,” “survminer,” and “timeROC.”

## Results

An overview of our study population of *n* = 203 individuals with baseline SCD is listed in Table 1. Significant group differences between the non-converted SCD group and MCI/AD converters were observed for CSF Aβ_42_ (***p <  0.001, Wilcoxon rank-sum test) (Table 1), plasma Aβ_42_ (*p* value 0.003), and Aβ_42/40_ ratio (*p* value = 0.002). Furthermore, significant differences were also found for APOEε4, MMSE and age (*p* value < 0.001, 0.036 and <  0.001).

**Table 1:** Baseline characteristics of the study population (*n* = 203) based on clinical diagnosis. Values are listed as the mean (± standard deviation) and dichotomous data as *n* (%)

	Total population ***n*** = 203		
	Non-converted SCD	SCD to MCI/AD	***p*** value
Characteristics	*n* = 180 (89%)	*n* = 23 (11%)	
Age, year	60 (±9)	67 (±8)	< 0.001
Female	74 (41%)	14 (61%)	0.072
MMSE	28 (±1)	28 (±1)	0.036
APOE ε4 carrier (reported)	61 (34%)	16 (70%)	< 0.001
ε4 homozygotes	51 (84%)	12 (75%)	–
ε4 heterozygotes	10 (16%)	4 (25%)	–
Follow-up duration, y	2.7 (±2.1)	–	–
Time to progression, y	–	2.5 (±2.2)	–
CSF Aβ_42_, pg/ml	1053 (±246)	800 (±203)	< 0.001
Plasma Aβ_40_, pg/ml	208 (± 36)	203 (±34)	0.346
Plasma Aβ_42_, pg/ml	10 (±2)	9 (±2)	0.003
Plasma Aβ_42/40_ ratio	49 (±7)	44 (±7)	0.002

Abbreviations: *MCI* mild cognitive impairment, *MMSE* Mini-Mental State Examination, *SCD* subjective cognitive decline

**Fig. 2:**
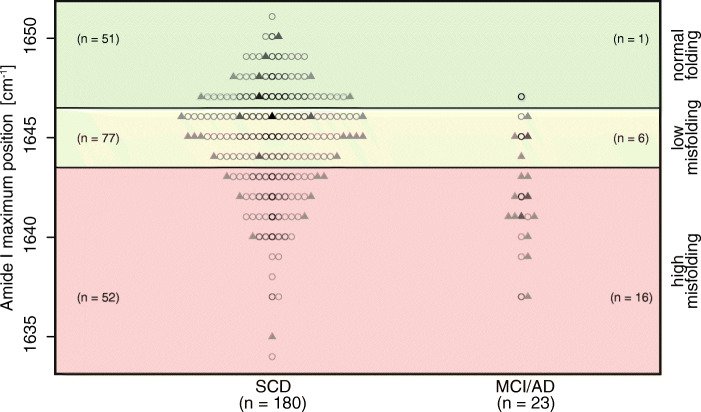
Read-out frequencies of the Aβ structure biomarker from the baseline plasma of 203 SCD subjects. Out of 180 non-converted SCD subjects, 51 were classified as having normal secondary structure distribution similar to that in the non-diseased subjects. They are above 1646 cm^− 1^ (green). Seventy-seven subjects showed low misfolding (i.e., ≤ 1646 cm^− 1^ and ≥ 1644 cm^− 1^) (yellow) and 52 had a high Aβ misfolding status (below 1644 cm^− 1^) (red). Out of 22 converters showing misfolding, 16 showed high misfolding and six converters showed low misfolding. The misfolding as a structure biomarker correctly predicted all 22 converters within the 6 years of follow-up. In subjects with normal misfolding, one converter was observed. This individual acquired the disease after 9.15 years. In addition, for each patient, the CSF status is displayed (● = normal; ▲ = abnormal), and the length of follow-up time is colored ascending from light gray to black (  = [≤ 2.5],  = [> 2.5–< 6.0],  = [≥ 6.0–< 9.0], ● = [≥ 9.0])

The structure-biomarker readout, the amide I band maxima, of all 203 participants is shown in Fig. 2 compared to the predefined cutoff values for low and high misfolding. Among the SCD subjects, who did not convert, 51 subjects displayed normal folding patterns (> 1646 cm^− 1^), the largest group (*n* = 77) showed low misfolding (≥1644 cm^− 1^ and ≤ 1646 cm^− 1^), and 52 subjects exhibited high misfolding (< 1644 cm^− 1^). In the group that had converted to MCI/AD within 6 years of follow-up, all 22 subjects showed already misfolding at baseline (indicated by red and yellow). A group of 16 of these 22 subjects had highly misfolded Aβ within a mean progression time of 2.2 ± 1.6 years, whereas the other 6 subjects who converted within 3.4 ± 2.2 years showed low Aβ misfolding. One subject that converted to AD was observed in the normal folding group above 1646 cm^− 1^ (green area). On post hoc inspection of this patient’s report, this individual converted not within the 6 years but after 9.15 years.

Furthermore, the mean progression time from SCD to MCI or AD-dementia was shorter when both the structure of Aβ and the decrease in CSF Aβ_42_ showed abnormal values. When Aβ misfolding and normal CSF Aβ_42_ were observed, the average progression time was 3.9 ± 2.8 years, but when Aβ misfolding and lower CSF Aβ_42_ were recorded, a much shorter progression time of 1.8 ± 1.2 years was observed.

The plasma Aβ structure biomarker correlated to the CSF Aβ_42_ biomarker, with *p* = 0.037 and *r* = 0.15, but not to the plasma Aβ biomarkers (supplementary Fig. S-6). In the converted group, 65% showed decreased CSF Aβ_42_, whereas in the non-converted SCD group, only 17% showed decreased CSF Aβ_42_ at baseline. The raw data shown in Fig. 2 largely overlap in the currently short mean follow-up time of 2.7 years. However, different individual follow-up times require an appropriate time-dependent statistical analysis.

To quantify the prognostic value provided by the structure biomarker to predict conversion to clinically diagnosed MCI or AD, we performed Cox proportional hazard analysis (Table 2), which takes into account the variability in follow-up times. If the biomarker frequency in cm^− 1^ was considered on a continuous scale within the covariate-adjusted Cox proportional hazard model, the risk for MCI or AD conversion increased by the factor of HR = 1.28 per cm^− 1^ decrease (95% CI 1.1–1.46, *p* = 0.0002). The HR for APOEε4, age, sex and the structure biomarker were also determined (Table 2). In the models adjusted for age, sex, and APOEε4, the group with high misfolding (< 1644 cm^− 1^) showed an HR of 19 (95% CI 2.249, 157.826), and the group with low misfolding (≥ 1644 cm^− 1^ and ≤ 1646 cm^− 1^) showed an HR of 11 (95% CI 1.048, 110.089) for future clinical conversion compared to the normal folding (> 1646 cm^− 1^) reference group.

**Table 2:** Cox proportional hazard models for Aβ misfolding when unadjusted and adjusted for the risk factors sex, age, and APOEε4. *HR* hazard ratio

	**Unadjusted model** **(Aβ misfolding)**		**Adjusted model** **(Aβ misfolding)**	
	HR (95% CI)	*p* value	HR (95% CI)	*p* value
APOE carrier	Not included		3.3 (1.3, 8.1)	0.01
Age	Not included		1.09 (1.02, 1.15)	0.008
Sex	Not included		1.8 (0.8, 4.5)	0.2
Aβ misfolding				
Low misfolding vs normal folding	4.6 (0.6, 38.5)	0.159	10.7 (1.05, 110.1)	0.046
High misfolding vs normal folding	14.0 (1.8, 105.7)	0.011	18.8 (2.2, 157.8)	0.007
Observations	203		203	
R^2^	0.073		0.159	
	**Adjusted model** **(Aβ**_**42/40**_ **ratio)**		**Full model** **(Aβ misfolding + Aβ**_**42/40**_ **ratio)**	
	HR (95% CI)	*p* value	HR (95% CI)	*p* value
APOE carrier	2.7 (1.1, 6.7)	0.034	3.0 (1.2, 7.4)	0.021
Age	1.05 (0.997, 1.113)	0.07	1.07 (1.0, 1.14)	0.049
Sex	1.6 (0.7, 3.8)	0.3	1.3 (0.5, 3.4)	0.6
Aβ misfolding	Not included			
Low misfolding vs normal folding			9.3 (0.9,91.1)	0.057
High misfolding vs normal folding			17.0 (2.1, 139.8)	0.009
Aβ_42/40_ ratio	0.9 (0.8, 0.99)	0.024	0.9 (0.87, 0.998)	0.046
Observations	203		203	
R^2^	0.120		0.178	

In an extended Cox proportional hazard analysis, we also included those with other forms of dementia (supplementary Table 1). Other forms of dementias were diagnosed in 3.3% of *n* = 210 subjects. Of these, 1.9% had frontotemporal dementia, 0.5% had vascular dementia, and 1.0% were not further defined. Since misfolding is a specific marker for AD, but does not differentiate between healthy individuals and other forms of dementias, data from those with the other dementias were censored in the model when diagnosed [26]. The hazard ratios were also 11 and 19 when including those with other forms of dementia (supplementary Table 1).

**Fig. 3:**
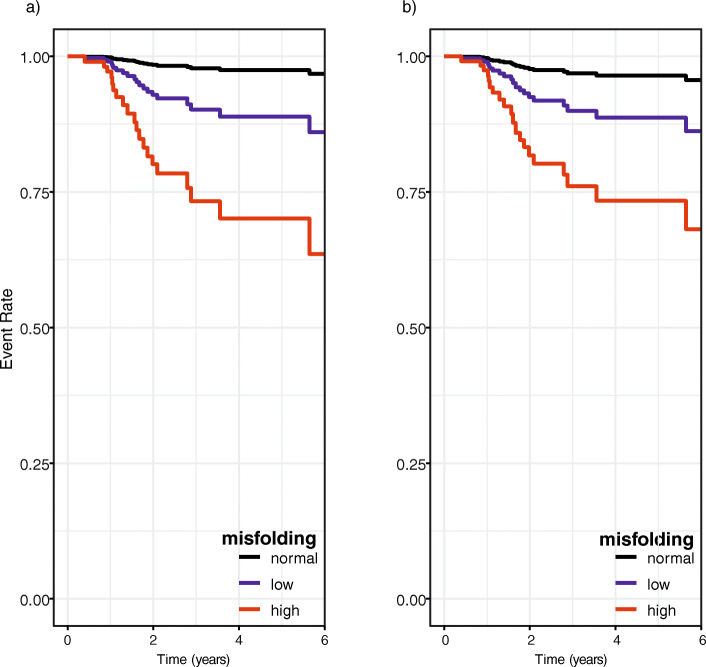
Kaplan-Meier survival curves at baseline to predict progression to clinically diagnosed MCI or AD using the structure biomarker. The unadjusted model based only on biomarker frequency is shown in **a** and an adjusted model including age, sex, and APOEε4 is shown in **b** for all three groups distinguished by the structure biomarker being < 1644 cm^− 1^ for high misfolding, ≥ 1644 cm^− 1^ and ≤ 1646 cm^− 1^ for low misfolding, and > 1646 cm^− 1^ for normal folding

The resulting Kaplan-Meier curves that visualize the probability of conversion within 6 years of follow-up for high misfolding, low misfolding, and normal folding are shown in Fig. 3 for the adjusted (a) and unadjusted (b) models. The three groups identified by the structure biomarker exhibited different levels of risk and differed in both models in contrast to the raw data shown in Fig. 2.

**Fig. 4:**
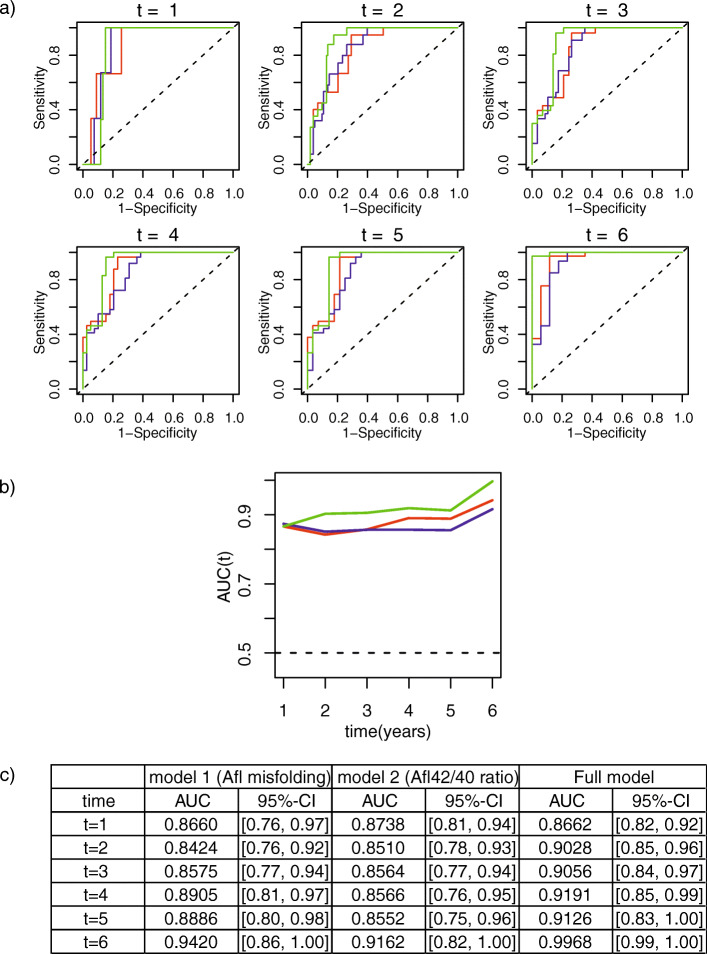
**a** Time-dependent ROC (t-ROC) curves for all three biomarker models. In addition to the structure biomarker, the risk factors age, sex, and APOEε4 status were also included (red). In addition, t-ROC analyses for the Aβ_42/40_ biomarker in plasma are shown in blue. Considering the risk factors and both plasma biomarkers in a panel, the AUC increased considerably with the risk biomarker panel (green). **b** T-ROC curve analysis overview for the 6-year follow-up period in each model. The time course of the structure biomarker, including the risk factors age, sex, and APOEε4, is shown in red. In blue, the Aβ_42/40_ ratio biomarker is shown, and the biomarker panel including risk factors and both plasma markers is shown in green. The biomarker panel shows an increase in performance compared to the plasma markers alone. **c** AUC values with confidence intervals for all models within 6 years. *Model 1* represents the performance of the structure biomarker (adjusted model), and *Model 2* shows the performance of the Aβ_42/40_ ratio and the risk panel including both plasma biomarkers in an adjusted model

In the next step, we quantified the accuracy of the structure biomarker to predict the conversion to clinically diagnosed MCI or AD-dementia by t-ROC analysis. The converters at each year of follow-up are shown in detail in Table 2 in the supplementary material. In addition to the structure biomarker frequency, the covariates of sex, age, and APOEε4 status were also considered. In Fig. 4a, the ROC curves for 6 years of follow-up are shown. For a better overview of the performance during the follow-up period, the two biomarkers and the biomarker panel are summarized in Fig. 4b. In Fig. 4c, the detailed values are listed. When including the covariates, the AUC for the structure biomarker (in red) was well above 0.84 for all 6 years and increased to 0.94 in the sixth year (Fig. 4). The Aβ_42/40_ biomarker performance is shown in blue; in green, the performance of the biomarker panel, which includes the structure biomarker, Aβ_42/40_ and age, sex, and APOEε4, is shown. The structure biomarker and Aβ_42/40_ perform similarly in the first 3 years, but visually, a better performance of the structure biomarker was observed in the last 3 years. Most notably, with the biomarker panel, the AUC (Fig. 4, in green) increased up to 0.997 for 6 years with a narrower confidence interval than the single biomarker.

## Discussion

In the present study, we validated the prognostic performance of Aβ misfolding as a structure biomarker by measuring the Aβ secondary structure distribution in baseline plasma samples from SCD subjects. The prognostic value of this biomarker for future conversion to clinical MCI and dementia due to AD was determined, adjusted for the covariates age, sex, and APOEε4. Additionally, we investigated whether the combination of different plasma biomarkers would increase the prognostic accuracy. For this purpose, we considered not only the misfolding of Aβ but also the plasma Aβ_42/40_ ratio level.

All 22 converters who developed MCI or dementia due to AD within 6 years of follow-up showed misfolding in the baseline plasma sample and thus could be correctly predicted. One converter who showed no misfolding at baseline converted later, that is, after 9.15 years. Moreover, this subject showed also no abnormal CSF Aβ_42_ at baseline. In this subject, increased Aβ misfolding in the plasma may have occurred after the baseline collection.

A large number of 52 SCD subjects with high Aβ misfolding had not yet converted and the converted and non-converted SCD subjects showed large overlap. These two groups might not have been differentiated in the raw data, as the average follow-up time in this cohort was only 2.7 years, which might be too short. Previous studies showed that our structure biomarker predicts conversion up to 14 years in advance [35]. In the ESTHER study, the average conversion time to AD-dementia was 8 years after baseline blood sample was taken [25]. In the current study, only those subjects with very rapid disease progression could be identified as converters. We hypothesize that subjects with high misfolding, but not subjects with normal folding (*n* = 51), will convert in the nearby future. The Amsterdam Dementia Cohort study is still ongoing, and it remains important for the future to repeat the statistical analysis with an extended follow-up period with the non-converted group.

To take this short follow-up time and the individual differences in follow-up and conversion times into account, an appropriate time-dependent statistical analysis was applied. This allowed us to determine the performance of the structure biomarker more accurately than by the raw data alone. Using this statistical analysis approach, the subjects with high misfolding showed a high risk, with an HR of 19, whereas those with low misfolding had a lower risk, with an HR of 11 compared to the normal folding group. Out of 22 converters, 16 showed high misfolding, whereas 6 converters showed low misfolding. The relative decrease in the readout indicated an increasing risk in the HR = 1.28 per cm^− 1^ decrease. Interestingly, converters with high misfolding of Aβ and an associated higher risk had a faster mean progression time (2.2 ± 1.6 years) than those with low Aβ misfolding (3.4 ± 2.2 years) and lower risk.

Kaplan-Meier survival curves in Fig. 3 demonstrated that the three groups (high misfolding, low misfolding, normal Aβ folding) diverged clearly from each other during the 6 years of follow-up (Fig. 3), which was in contrast to the raw data (Fig. 2). To quantify the accuracy of the prognosis by the structure biomarker, additional time-resolved ROC curves were determined, yielding high AUCs between 0.84 and 0.94 dependent on the length of follow-up time.

Furthermore, we investigated whether the combination of different plasma biomarkers could increase the prognostic precision. For this purpose, we considered not only misfolding of Aβ but also the plasma Aβ_42/40_ ratios measured by SIMOA technology [15]. The performance of the ratio biomarker was analyzed with the same advanced statistical approach as the structure biomarker and provided similar prognostic values. Most remarkably, the combination of both plasma biomarkers provided an added value with a significantly increased AUC to 0.99 (95% CI 0.99–1.00) for a follow-up period of 6 years. Additionally, it also provided a narrower confidence interval compared to models containing only one of the two markers. The biomarker panel can thus provide a very high prognostic accuracy to predict conversion to MCI or dementia due to AD using plasma biomarkers and covariates only.

The added value provided by the panel might be explained by different biological processes that the biomarkers reflect. Aβ_42/40_ primarily represents amyloid plaque formation, as this biomarker is highly associated with Aβ-PET scans and amyloidosis [15, 48]. The structure biomarker, on the other hand, monitors the misfolding into β-sheet-enriched structures, which is assumed to occur many years before plaque formation. In this study, Aβ misfolding correlated with the Aβ_42_ decrease in CSF, but did not correlate with plasma Aβ (see supplementary Fig. S-6). In our former studies, we already reported correlations between the structure-based biomarker in plasma and the Aβ_42_ decrease in CSF for prodromal to severe AD disease stages [24–26]. However, the structure-based biomarker does not correlate with the decrease of Aβ_42_ in plasma as detected with the iRS in the current study. This might be explained by the early disease state of these patients at baseline. In CSF, the Aβ_42_ concentration seems to be more sensitive to earlier changes in the brain than the concentration in plasma, which might explain the low correlation with the structure biomarker in plasma. These findings rather support the hypothesis that misfolding of Aβ is one of the initial events in AD development preceding Aβ accumulation in the brain (PET), which reduces the concentration of Aβ_42_ in CSF and plasma. The structure biomarker would therefore indicate an earlier stage of disease development than the Aβ concentration in plasma. In conclusion, it seems that by combining both plasma biomarkers, a broader time window can be monitored, starting with initial Aβ misfolding and the subsequent amyloid formation. Thus, improvements in prognostic accuracy were achieved when taking both plasma biomarkers into account. Interestingly, the mean conversion time from SCD to MCI or AD-dementia was shorter when both the structure biomarker and the concentration of Aβ_42_ in CSF showed abnormal values.

In addition to the potential for monitoring different disease-related biological processes, the use of multiple biomarkers might compensate for method-based biases as well. Changes in Aβ concentrations, e.g., due to the circadian cycle, due to an initial rise in liquid Aβ levels in presymptomatic individuals or due to other diseases, could affect the concentration-dependent measurement of SIMOA technology. iRS measurements, in contrast, should be more robust to concentration changes due to biological variation since SIMOA focuses on the ratio between individual conformational species of Aβ rather than on the absolute concentration of selected Aβ isoforms (e.g., Aβ_1–42_, Aβ_1–40_). However, misfolding of Aβ may be impaired by cross-seeding of other amyloidogenic proteins, e.g., in the presence of type II diabetes leading to AD-independent conformational changes, which in turn affects the iRS readout but not SIMOA results.

Among the strengths of our study is that we stratified risk groups for SCD patients to convert to clinical MCI or AD within the following 6 years by using only plasma biomarkers. While the previously measured ESTHER cohort is a prospective community-based cohort of older adults, the SCIENCe cohort measured here includes help-seeking individuals in a memory clinic setting. Since cognitive impairment can also be associated with other causes, such as mood states, stress or other neurological disorders, it is important to identify a biomarker that can identify SCD patients in a preclinical AD state. In the ESTHER cohort, we were able to show that the structural biomarker correlates with the future development of AD [25]. For SCD patients, a simple blood screening may be a great benefit. The earlier changes caused by AD can be detected, the more adequately the affected persons can be cared for and treated. In contrast to patients who already have objectively measurable cognitive deficits, SCD patients do not yet exhibit AD-associated irreversible brain damage. Early testing procedures, which can easily be performed on blood as routine and regular testing, can be used to initiate early countermeasures, including nontherapeutic countermeasures such as lifestyle changes, to help patients improve mentally and physically. On the other hand, patients who seem to have no risk can be reassured for the time being.

The Amsterdam Dementia Cohort study is still ongoing, and therefore, it remains important for the future to repeat the statistical analysis with an extended follow-up time with the non-converted group. To confirm the current analysis, it would be important to see that the groups significantly differ not only in the statistically analyzed data but also in the raw data. The broad overlap in the raw data is a weakness of the study, as the average follow-up time is currently too short. Furthermore, an additional external validation study with larger numbers must be performed to validate the findings here.

## Conclusion

In summary, this study revealed that a panel of structure- and concentration-based Aβ plasma biomarkers precisely predicts conversion to clinical MCI and dementia due to AD 6 years in advance in individuals with SCD. It provides an earlier time window for screening high-risk symptom-free subjects for potential AD treatments. The proposed plasma biomarker panel including covariates offers a less invasive and cost-effective alternative to currently used CSF biomarkers and PET scanning.

## Supplementary Information

**Additional file 1: Supplementary Table 1.** Cox proportional hazard regression models including other forms of dementia.

**Additional file 2: Supplementary Table 2.** Risks and events by year with amide I maximum ≥1644 cm^− 1^, < 1644 cm^− 1^, and all subjects (*n* = 203).

**Additional file 3: Supplementary Figure S-1.** HJ 5.1 binding characteristics. The antibody recognizes Aβ monomers derived from chicken telencephalonic cells with an amide I maximum of 1653 cm^− 1^ (A) and Aβ_1–42_ fibrils with a maximum of 1628 cm^− 1^ (B). CSF from healthy individuals yielded a maximum of 1645 cm^− 1^ (C) above the threshold and for AD subjects a maximum of 1640 cm^− 1^ below the threshold. (D).

**Additional file 4: Supplementary Figure S-2.** Reproducibility of plasma measurements and specific binding of Aβ with HJ 5.1. (A) Repeated analysis of the same antibody-depleted plasma sample with HJ 5.1 results in the same read out at 1642 cm^-1^. (B) A control measurement of an Aβ-depleted sample shows no signal at all, indicating no unspecific binding of plasma proteins.

**Additional file 5: Supplementary Figure S-3.** Depletion of plasma samples provide the same readout. (A) The same plasma sample shows the same spectra for both capture antibodies, HJ 5.1 (depleted sample, black) and A8978 (non-depleted sample, dotted line) indicating that both antibodies extract the same Aβ fractions out of the plasma sample and providing the same read out at 1640 cm^-1^. (B) Single depletion (dotted line) and double depletion (grey) of a sample containing few amounts of human antibodies results in the same read-out when measured with HJ 5.1 as with A8978 (black). (C) A different non-depleted plasma sample with a higher amount of antibodies is dominated by antibodies absorbing at 1638 cm^-1^ and a shoulder at 1685 cm^-1^ (grey). For comparison an antibody spectrum is shown in grey with a dotted line. Single depletion of a plasma sample containing a large amount of human antibodies still contains antibody contributions (blue). However, the double-depleted sample shows no longer antibody contribution (black, dotted line). The read-out is again the same as with the A8978 (black). All depleted samples underwent a quality control to ensure complete removal of human IgGs.

**Additional file 6: Supplementary Figure S-4.** No correlation could be observed between amide I maximum and age of the samples (rs= 0.01, *p*-value = 0.87).

**Additional file 7: Supplementary Figure S-5.** Binding of Aβ from plasma and CSF samples with HJ 5.1 as the capture antibody. (A) Amide I signal from a plasma sample of an SCD patient who did not convert (grey) and an SCD patient who did convert during the follow-up time (black). Amide I maxima varied from 1647-1651 cm^-1^ during circulation due to unbound plasma proteins that interfere with the signal. During washing, only the Aβ signal is left, showing constant and differentiable amide I maxima. (B) Amide I signal from a CSF healthy control sample (grey) and an AD subject (black). The signal stays stable during circulation and washing, indicating no change in the conformational structure of bound Aβ. Less interfering proteins in CSF samples did not influence the signal.

**Additional file 8: Supplementary Figure S-6.** Correlation plots between CSF and plasma levels of Aβ and amide I maximum frequency. The appropriate biomarker is presented on the y-axis, the amide I maximum position on the x-axis and the spearman rank value (r) and p-value above the distribution points, equally. SCD subjects are plotted in blue, MCI subjects in orange and AD subjects in red. Biomarker cutoffs are shown as dashed lines. The structure-based biomarker showed weak correlation with CSF Aβ_42_ but not with the concentration biomarkers in plasma (Aβ_40_, Aβ_42_ and Aβ_40/42_).

## Data Availability

The datasets generated and analyzed during the current study are not publicly available due to local privacy regulations but are available from the corresponding author on reasonable request.

## Abbreviations

AD: Alzheimer’s disease
Aβ: Amyloid-β
AUC: Area under curve
CSF: Cerebrospinal fluid
SCD: Subjective cognitive decline
MCI: Mild cognitive impairment
APOE: Apolipoprotein E
SIMOA: Single molecule array
t-ROC: Time-dependent receiver operating characteristic
MMSE: Mini-Mental State Examination
HR: Hazard ratio
PBS: Phosphate-buffered saline
IR: Infrared
CI: Confidence interval
PET: Positron emission tomography
OD: Other dementia
