# Correction to: Amyloid-β misfolding as a plasma biomarker indicates risk for future clinical Alzheimer’s disease in individuals with subjective cognitive decline

**DOI:** 10.1186/s13195-021-00770-2

**Published:** 2021-01-15

**Authors:** Julia Stockmann, Inge M. W. Verberk, Nina Timmesfeld, Robin Denz, Brian Budde, Julia Lange-Leifhelm, Philip Scheltens, Wiesje M. van der Flier, Andreas Nabers, Charlotte E. Teunissen, Klaus Gerwert

**Affiliations:** 1 Competence Center for Biospectroscopy, Center for Protein Diagnostics (PRODI), Ruhr-University Bochum, Bochum, Germany; 2 Department of Biophysics, Faculty of Biology and Biotechnology, Ruhr University Bochum, Bochum, Germany; 3 Department of Clinical Chemistry, Neurochemistry Laboratory, Amsterdam Neuroscience, Vrije Universiteit Amsterdam, Amsterdam UMC, Amsterdam, The Netherlands; 4 Department of Neurology, Alzheimer Center Amsterdam, Amsterdam Neuroscience, Vrije Universiteit Amsterdam, Amsterdam UMC, Amsterdam, The Netherlands; 5 Department of Medical Informatics, Biometry and Epidemiology, Ruhr University Bochum, Bochum, Germany

**Correction to: Alz Res Ther (2020) 12:169**

**https://doi.org/10.1186/s13195-020-00738-8**

Following publication of the original article [1], the authors noticed that the published figures have errors which was occurred during processing of the figures in production team.

1. fonts are shifted (Figure 1)
2. colors are not displayed (Figure 2, open circles should be colored)
3. labelling is incorrect (Figure 4, "Afl" should be "Aß", Supplementary Figures "D Absorbance" should be "Δ absorbance" (Δ = delta))

The correct Figures 1, 2 and 4 are shown below. The original article [1] has been updated.

**Figure 1:**
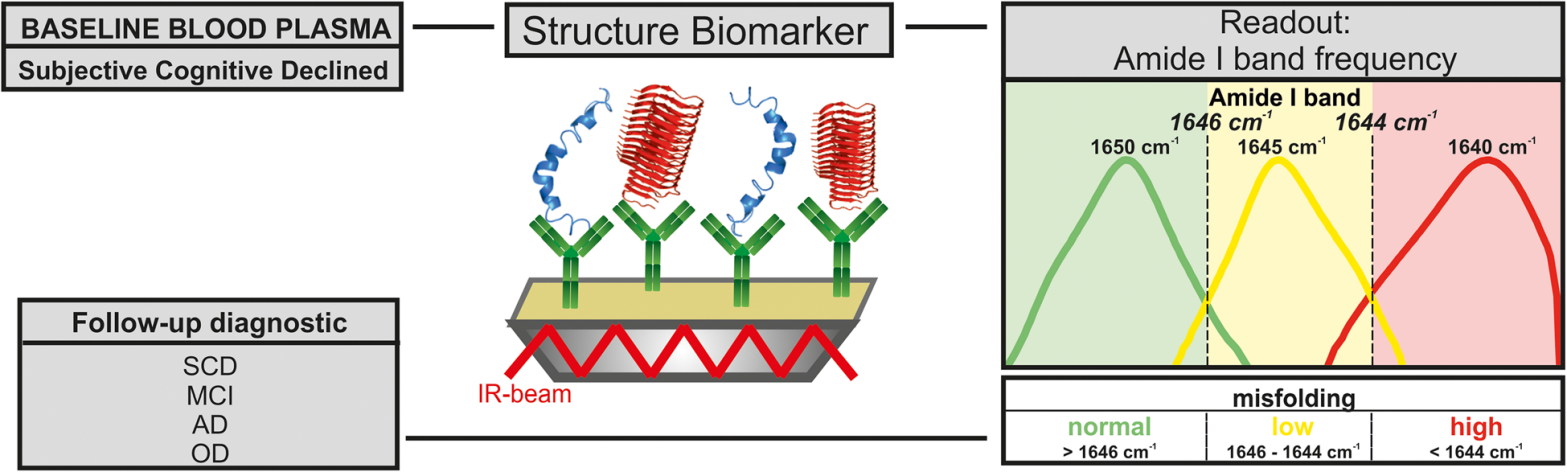
Fig. 1

**Figure 2:**
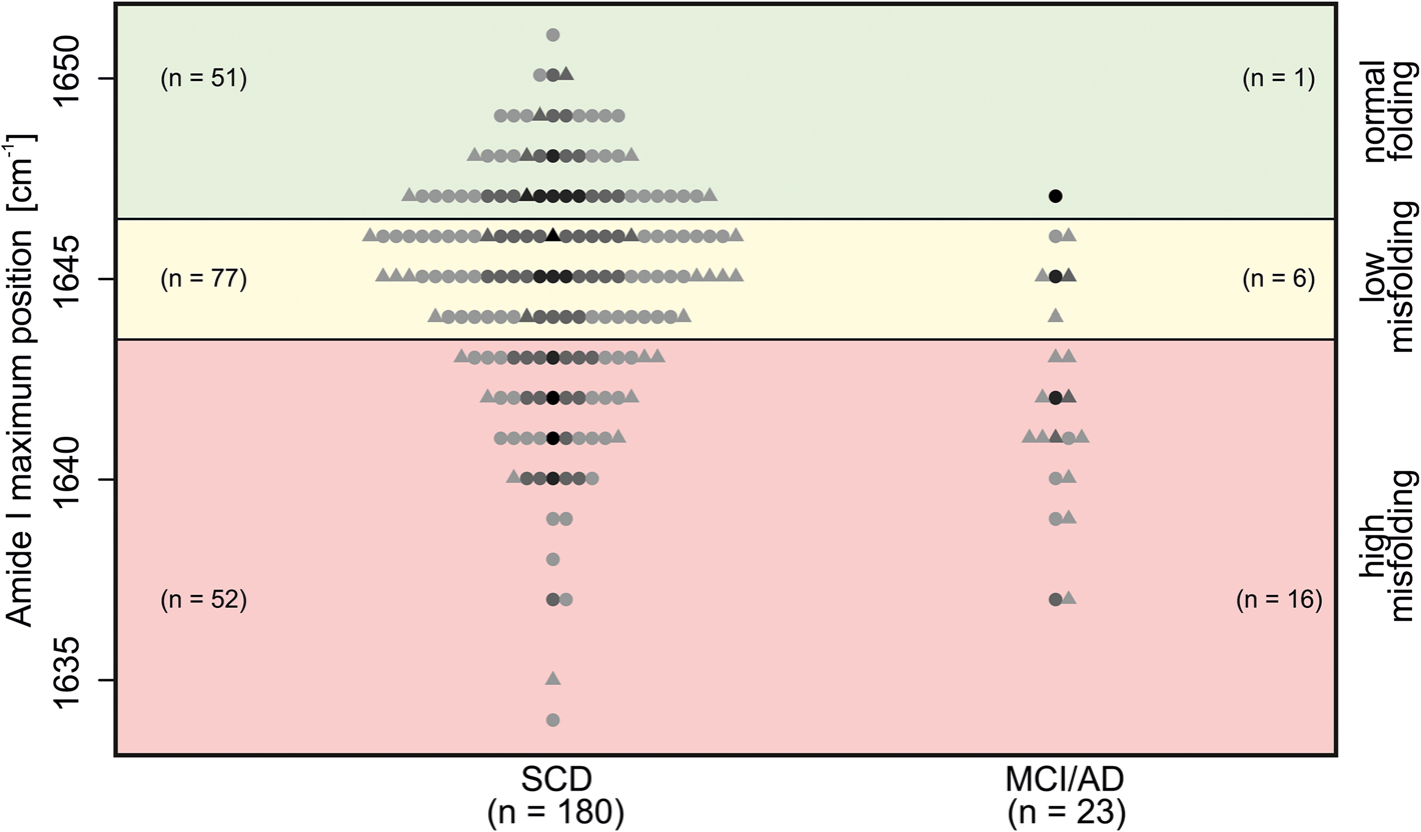
Fig. 2

**Figure 3:**
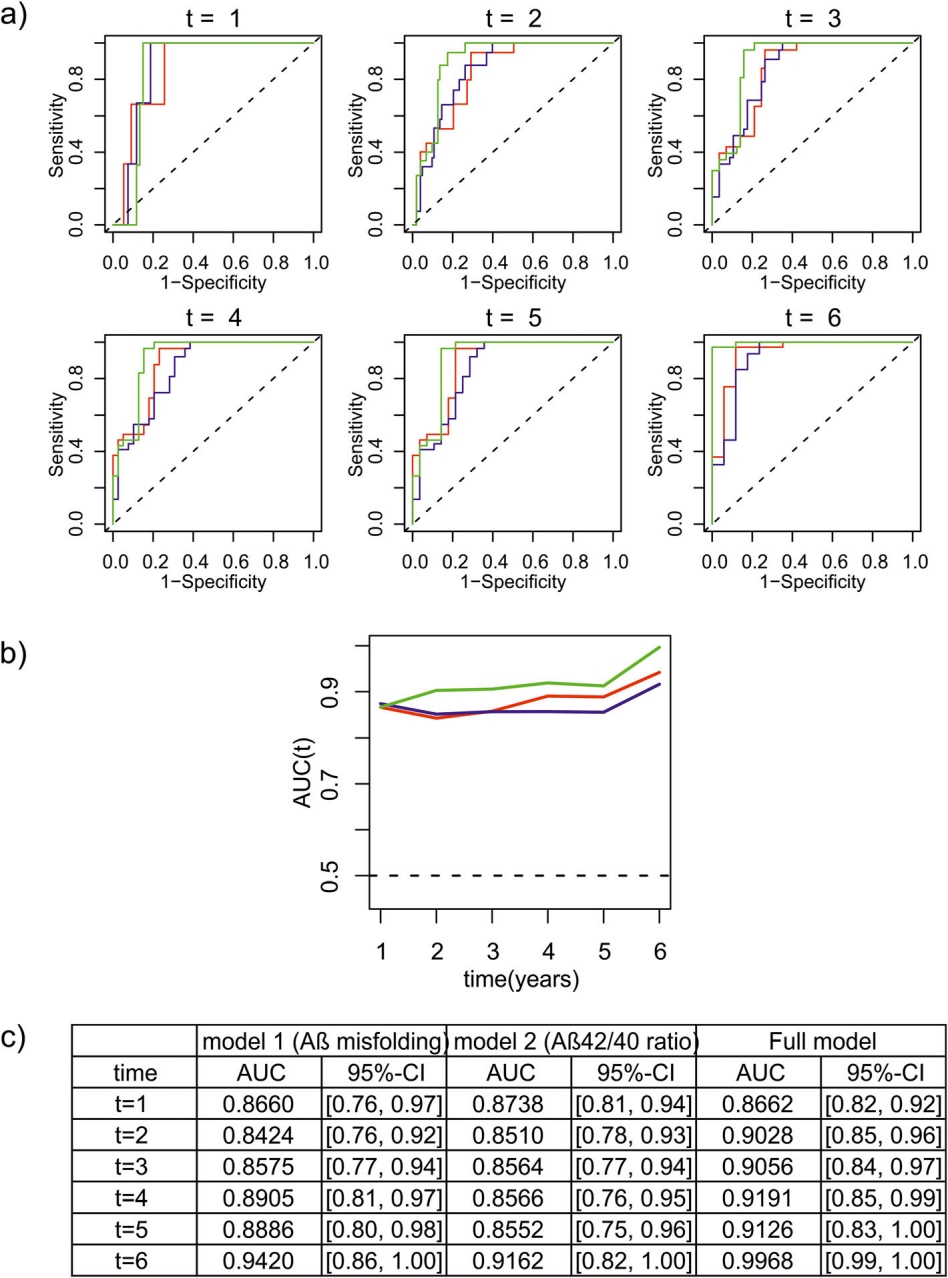
Fig. 4
